# Chemical Informatics Combined with Kendrick Mass Analysis to Enhance Annotation and Identify Pathways in Soybean Metabolomics

**DOI:** 10.3390/metabo15020073

**Published:** 2025-01-24

**Authors:** Troy D. Wood, Erin R. Tiede, Alexandra M. Izydorczak, Kevin J. Zemaitis, Heng Ye, Henry T. Nguyen

**Affiliations:** 1 Department of Chemistry, University at Buffalo, State University of New York, Buffalo, NY 14260-3000, USA; erintied@buffalo.edu (E.R.T.); kevin.zemaitis@pnnl.gov (K.J.Z.); 2 Division of Plant Sciences and National Center for Soybean Biotechnology, University of Missouri, Columbia, MO 65211, USA; yehe@missouri.edu (H.Y.); nguyenhenry@missouri.edu (H.T.N.)

**Keywords:** Kendrick mass defect, metabolomics, soybean, electrospray ionization, drought, FT-ICR, chemical informatics

## Abstract

Background: Among abiotic stresses to agricultural crops, drought stress is the most prolific and has worldwide detrimental impacts. The soybean (*Glycine max*) is one of the most important sources of nutrition to both livestock and humans. Different plant introductions (PI) of soybeans have been identified to have different drought tolerance levels. Objectives: Here, two soybean lines, Pana (drought sensitive) and PI 567731 (drought tolerant) were selected to identify chemical compounds and pathways which could be targets for metabolomic analysis induced by abiotic stress. Methods: Extracts from the two lines are analyzed by direct infusion electrospray ionization Fourier transform ion cyclotron resonance mass spectrometry. The high mass resolution and accuracy of the method allows for identification of ions from hundreds of different compounds in each cultivar. The exact *m*/*z* of these species were filtered through SoyCyc and the Human Metabolome Database to identify possible molecular formulas of the ions. Next, the exact *m*/*z* values were converted into Kendrick masses and their Kendrick mass defects (KMD) computed, which were then sorted from high to low KMD. This latter process assists in identifying many additional molecular formulas, and is noted to be particularly useful in identifying formulas whose mass difference corresponds to two hydrogen atoms. Results: In this study, more than 460 ionic formulas were identified in Pana, and more than 340 ionic formulas were identified in PI 567731, with many of these formulas reported from soybean for the first time. Conclusions: Using the SoyCyc matches, the metabolic pathways from each cultivar were compared, providing lists of molecular targets available to profile effects of abiotic stress on these soybean cultivars. Key metabolites include chlorophylls, pheophytins, mono- and diacylglycerols, cycloeucalenone, squalene, and plastoquinones and involve pathways which include the anabolism and catabolism of chlorophyll, glycolipid desaturation, and biosynthesis of phytosterols, plant sterols, and carotenoids.

## 1. Introduction

Abiotic stresses to agricultural crops can have a significant impact on crop yields, with drought being one of the most prolific [1]. Legumes such as soybean (*Glycine max*) are particularly susceptible to drought stress during growth stages, which has dramatic impacts on crop yield [2]. Reduced yields due to drought are the result of alterations in homeostasis, impacting composition of plant tissues at the molecular level [3]. Recent advances in agronomy have led to the identification of slow canopy wilting (SW) phenotypes in soybean which exhibits a tolerance to drought stress [4]. The plant introduction (PI) PI 567731, an exotic soybean germplasm, has been shown to consistently possess the SW phenotype and utilizes less water (and has less yield reduction) under drought conditions [4,5]. Unfortunately, many of the underlying metabolomic mechanisms for the drought tolerance of the PI 567731 are not clear [6]. Current understanding of the mechanisms by which certain cultivars have increased resistance to abiotic stress would be enhanced by knowledge of the molecular composition of components of soybean plants. Therefore, there is a need for analytical methods that can increase the number of metabolites identified.

Mass spectrometry (MS) is a rapidly growing technique for fingerprinting in soybean metabolomics [7,8]. Part of this growth is due to the superior sensitivity of MS and structural elucidation of components afforded by tandem mass spectrometry (MS/MS), which can be enhanced further by combination with chromatography methods. Furthermore, MS can be used to identify metabolites as a function of plant tissue, growth stage, or in response to abiotic stress. A report which examined metabolic profiles of soybean leaves using gas chromatography–mass spectrometry (GC-MS) and liquid chromatography–mass spectrometry (LC-MS) found that several important pathways related to nitrogen and sugar metabolism were impacted by drought and heat stresses [3]. Another study used GC-MS and ultrahigh performance liquid chromatography (UPLC) to identify more than 160 metabolites from seeds of different soybean cultivars [9]. The impact of flooding stress on soybean plants was investigated by capillary electrophoresis coupled to MS, revealing numerous metabolites sensitive to flooding, including increased levels of gamma-aminobutyric acid, glycine, NADH2, and phosphoenol pyruvate [10]. Soybean metabolomics in the context of drought stress was investigated using UPLC and tandem mass spectrometry, and indicated that amino acid metabolism and lipid metabolism play roles in drought resistance [11]; in particular, the tricarboxylic acid (TCA) cycle was identified as a core pathway that either enables drought resistance or is the consequence of drought tolerance [11]. Molecular tomography has been used as a metabolomics approach to probe the symbiotic interaction between soybeans and their root nodules using mass spectrometry imaging, information which could be used to help improve crop yields through an understanding of plant–microbe processes and the metabolic roles nodules play for soybean nitrogen use [12]. Our group has promoted the utility of high-resolution mass spectrometry, independent of chromatographic methods, for metabolomic analysis of soybeans, showing clear distinction of soybean leaves due to senescence [13] and for drought-stressed soybean leaves, particularly with respect to chlorophyll and its related metabolites [14]. Mass spectrometry as an analytical platform for soybean metabolomics induced by abiotic stress is particularly useful as it can be utilized for investigation across anatomy, including underused parts of the plant [15].

Chromatographic methods in combination with mass spectrometry (e.g., GC-MS and LC-MS) are used widely in metabolomics. However, it is also important to recognize that high mass resolving power and high mass accuracy measurements make it possible to assign molecular formulas to components in very complex mixtures, even when chromatography is not used. Methods to assist in molecular formula identification are of paramount importance. Kendrick recognized that by rescaling a mass spectrum from the IUPAC mass scale (^12^C is exactly 12 Da) to a mass scale based on methylene units enables ready identification of a homologous series of compounds of the same class and type, but with different extents of alkylation [16]. Effectively, the IUPAC mass is converted into a Kendrick mass:

(1) Kendrick mass = IUPAC mass × (14/14.01565)

By rescaling the mass spectrum, compounds with identical numbers of heteroatoms and ring double bond equivalents (RDBE) possess identical Kendrick mass defects (KMD):

(2) KMD = (nominal Kendrick mass − exact Kendrick mass)

Obtaining KMD has shown great utility in identification of molecular formulas from highly complex mixtures of hydrocarbons [17,18,19,20,21], synthetic polymers [22,23,24,25,26,27,28,29,30], and specimens of biologic origin for metabolomics [31,32,33,34,35,36,37,38]. A lipidomic analysis used KMD in combination with liquid chromatography and high-resolution mass analysis to annotate numerous classes of polar lipids from glioblastoma cell cultures; this report also identified short-chain sulfatides as being the most highly modulated class of polar lipids [31]. A method to rapidly determine lipid classes used referenced Kendrick mass defect (RKMD) by converting lipid masses to the Kendrick mass scale, followed by referencing each converted mass to each lipid class; this method was applied to identification of components within a lipid extract of bovine milk [32]. KMD [37] and the related multidimensional stoichiometric compound classification (MSCC) method [39] have been employed for phytochemical assessment within plants.

Expanding upon an earlier study using direct infusion electrospray ionization (ESI) Fourier transform ion cyclotron resonance (FT-ICR) mass spectrometry to examine phytochemical composition of different soybean cultivars [14], here we more fully characterize the library of metabolites that are present in each cultivar and potential pathways involved. Measured *m*/*z* values were filtered through the SoyCyc database (https://soycyc.soybase.org/ (accessed on 10 December 2024)), populated with data from SoyBase (https://soybase.org (accessed on 10 December 2024)). [40] and the Human Metabolome Database (HMDB) (https://hmdb.ca/ (accessed on 10 December 2024)). [41], for initial assignments of molecular formulas. Subsequent KMD analysis was conducted to assign additional molecular formulas. Matches from the SoyCyc database were used for metabolic pathway mapping analysis, providing lists of molecular targets available to profile effects of abiotic stress. The impact of these findings on our previous study, which examined the role of watering as an abiotic stressor of the leaf metabolomes of these two cultivars [14], is discussed.

## 2. Materials and Methods

### 2.1. Materials

HPLC grade methanol was from Sigma-Aldrich (St. Louis, MO, USA; Cat. no.: 439193). For vacuum filtration of particulate matter, Cytiva Whatman filtration papers with an 11-micron pore size (Little Chalfont, Buckinghamshire, UK, Cat. 1001-055) were used.

Two cultivars of soybean (*Glycine max*) were grown in the field at the University of Missouri: the drought-sensitive cultivar Pana and the drought-tolerant cultivar PI 567731. Here, plants were grown for seven weeks in the field under normal irrigation conditions, with watering two days prior to tissue collection, when plants reached the R2 growth stage.

### 2.2. Extraction and Sample Preparation

Leaves from the two cultivars were collected from 20 plants/cultivar and flash frozen immediately following tissue collection and transported to the University of Missouri. Afterward, they were placed on dry ice and shipped to the University at Buffalo. There, the specimens were stored in polycarbonate Petri dishes at −20 °C until extractions were performed. Flash frozen leaves from multiple plants of each cultivar were pooled. Next, each group was individually macerated manually for five minutes in methanol using mortar and pestle. To remove particulates, vacuum filtration was performed. The samples were subsequently dried in a vacuum oven, then the dried residue was reconstituted into 2 mL of HPLC grade methanol. These samples were diluted by 50× prior to ESI FT-ICR analysis.

### 2.3. ESI FT-ICR Mass Spectrometry

Direct infusion ESI FT-ICR mass spectrometry was conducted using three replicates from each cultivar, the details of which are described previously [14]. Positive ion mode ESI was used because we have found high variability in negative ion mode ESI of soybean leaf extracts using polar solvents [13]. Once the mass spectra were collected, the data sets were processed as follows using Bruker Daltonics (Bremen, Germany) Data Analysis 4.0 software. Software was instructed to find all peaks with a signal-to-noise ratio ≥ 3 to produce a peak list. Next, the peak list was subjected to the deconvolution process, such that isotopic envelopes were determined and each individual ionic species was then grouped as part of the given isotopic cluster. A threshold of 0.1% peak area relative to the most intense peak (*m*/*z* 1073.506 in each cultivar list, corresponding to ion C_67_H_94_NaN_4_O_6_) was used. The peak list was reduced to the monoisotopic isotope of each isotopic cluster, and this was the *m*/*z* value used in compiling lists for each cultivar.

### 2.4. Data Processing and Analysis

Although the high-resolution analysis afforded by FT-ICR is useful in identifying potential elemental compositions, at higher *m*/*z* there may be multiple candidates within 3 ppm; thus, it is not obvious if one (or more) predicted elemental compositions is correct. To increase confidence in assignments of elemental compositions, a combined chemical informatics searching and Kendrick mass defect analysis procedure was used. After compilation of the *m*/*z* list for each cultivar, it was first passed through the SoyCyc database of metabolites (https://soycyc.soybase.org/ (accessed on 10 December 2024)); matches of either protonated, sodiated, or potassiated ions to the known metabolites within 3 ppm mass error was considered a confirmation of the ionic formula. Each list was then filtered through HMDB to discover matches to either protonated, sodiated, or potassiated ions in the database. For endogenous compounds, the 3 ppm mass error was again used to constitute a match. For non-natural compounds, however, a stricter limit of 1 ppm was used to constitute a match between the database and the *m*/*z* list. To further annotate the *m*/*z* with ionic formulas, each list was converted to the corresponding Kendrick mass and KMD calculated for each ion; ions were then sorted by KMD and plotted as nominal Kendrick mass vs. KMD to assist in identification of ionic formulas to those *m*/*z* which did not yet have one. Final lists of ionic formulas from each cultivar were then recorded and compared.

For those *m*/*z* values which matched entries in the SoyCyc database, an examination of the metabolic pathways involved was also performed to obtain context on how the cultivars might respond to drought at a molecular level. Note that the absence of an annotated peak in the list does not mean that metabolite is not present; rather, the metabolite is not detected with an abundance greater than 0.1% within the restrictive mass accuracy window employed. Metabolites from each cultivar identified in SoyCyc were the inputs into the Pathway Covering tool (https://pmn.plantcyc.org/cmpd-pwy-coverage.shtml (accessed on 10 December 2024)) using a constant cost function; the tool then computed a minimal-cost set of metabolic pathways for *Glycine max* from each cultivar’s data set. For this analysis, Pathway Tools version 26.0 [42] was used employing data identified within the SoyCyc 10.0.2 database.

## 3. Results and Discussion

### 3.1. ESI-FTICR of Soybean Leaf Extracts

**Figure 1:**
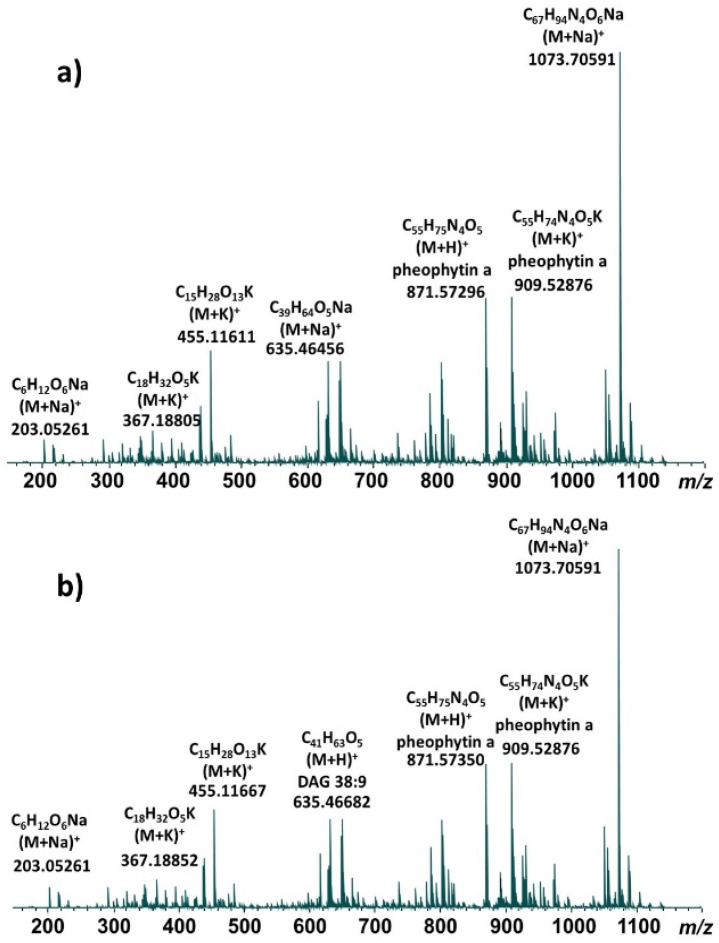
Direct infusion ESI FT-ICR mass spectra of methanolic soybean leaf extracts of cultivars (**a**) Pana and (**b**) PI 567731.

Representative direct infusion ESI FT-ICR mass spectra of methanolic leaf extracts are shown for the Pana cultivar (Figure 1a) and the PI 567731 cultivar (Figure 1b). The major components are highly similar for both cultivars, and the same base peak is observed for each at *m*/*z* 1073.706; our previous study indicates this molecule is derived from the sodiated ionic species of pheophytin *a* possessing an additional C_12_H_20_O moiety [14]. All of the detected ions are singly-charged. After deconvolution of each mass spectrum in Data Analysis 4.0, a total of 612 distinct isotopic clusters (Supplementary Table S1) were identified for the Pana methanolic extract, while 528 distinct isotopic clusters (Supplementary Table S2) were identified for the PI 567731 methanolic extract. Lists of *m*/*z* values using the monoisotopic peak for each cluster were compiled for each cultivar for subsequent comparison with databases and KMD analysis.

### 3.2. Data Processing to Identify Matches in SoyCyc

The *m*/*z* peak list from each cultivar was initially passed through the SoyCyc database to find potential matches to known constituents within 3 ppm mass error. With the Pana cultivar, *m*/*z* values were matched to protonated, sodiated, or potassiated ions from known soybean metabolite components in SoyCyc; ionic formulas were assigned based on the matches, and a mass error calculated. For the PI 567731 cultivar, *m*/*z* values matched protonated, sodiated, or potassiated ions of known soybean metabolites in SoyCyc. Ionic forms of metabolites detected exclusively in methanol extracts of Pana leaves are listed in Table 1, while ionic forms of the metabolites detected exclusively in methanol extracts of PI 567731 leaves are listed in Table 2.

Leaf extracts from the two cultivars share many ionic formulas that were matched. Prominent amongst these are mono- and diacylglycerols, pheophytin *a*, chlorophyll *a*, monosaccharides, disaccharides, xanthins, and vicenin-2 (a flavonoid diglucosylation product). Notable also is the simultaneous presence of plastoquinone, detected with products echinone and plastoquinol, essential components of photosynthetic electron transfer. Likewise, ubiquinol-8 and -9 were detected along with 3-demethylubiquinol-9 and demethylmenaquinol-8, key components of aerobic respiration and photosynthethic electron transfer. The metabolite cycloeucalenone is involved in phytosterol biosynthesis. Glutathione disulfide, the oxidized form of glutathione monomer, was also detected in both cultivar leaf extracts.

Pana is a drought-sensitive soybean cultivar. Using the known soybean metabolites identified in Table 1, there were several molecules from the class of carboxylic acids present in Pana that were not detected in PI 567731; these are essential precursors to lipids. Carlactone is an oxidation product of cartenal, possibly indicating oxidative stress in Pana, even in the control which has not experienced drought. This is further supported by the presence of glutathione disulfide (although this metabolite is also detected in the PI 567731 extracts). Galactopinitols are required substrates and products of galactosylcyclitol biosynthesis. The compound 15-*cis*-phytoene is needed for production of plastoquinol and carotenes. Likewise, menoquinol-8 is a polyprenyl quinone required for electron transport. A more diverse complement of pheophytins and chlorophylls are detected in Pana in comparison to PI 567731 (e.g., chlorophyll *b* was only detected in Pana). However, our earlier work showed that PI 567731 maintains greater levels of pheophytins and chlorophylls during drought [14].

In contrast, PI 567731 is a drought-tolerant soybean cultivar. As shown in Table 2, five such metabolites are detected in the methanolic extract of PI 567731 leaves. The galactosyl glycerol compound is 3-β-D-galactosyl-sn-glycerol, formed from the degradation of diacyl glycerols. Soyasapogenol B is a key precursor in the formation of its glucuronide. There are many possible structures for the trisaccharides, so anabolism of more complex saccharides from mono- and disaccharides might explain the appearance of trisaccharides here. Plastoquinones are electron carriers that are necessary building blocks for plastoquinol, and are found in chloroplasts, thus playing a central role in the photosynthetic electron transport chain.

### 3.3. Kendrick Mass Defect (KMD) Analysis

Although soybean metabolites are not human metabolites, it is recognized that they may share molecular formulas with common human metabolites. It is commonly recognized that plant metabolomes possess far greater molecular diversity than most organisms [43]. Indeed, collectively plants produce an estimate of 100,000 to 1 million metabolites, many of which play roles in resistance or tolerance to stressors, both biotic and abiotic in nature [43]. Here, the peak list for each soybean leaf cultivar was imported into HMDB and searched against possible matches to protonated, sodiated, or potassiated molecular formulas with known components of the human metabolome and exposome. In each case ionic formulas were matched within 3 ppm; in a few cases where a metabolite was not natural in humans, and is instead due to contact with a component in the environment, and therefore part of the human exposome, a stricter setting of 1 ppm mass error was tolerated to be a match. It should be noted that the vast majority of matches indicated less than 1 ppm error. This analysis yielded more than 300 matches between *m*/*z* and ionic formula for each cultivar.

**Figure 2:**
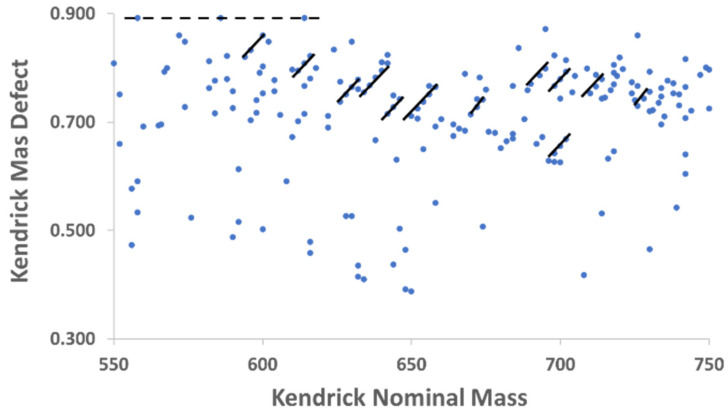
Kendrick nominal mass plot from 550–750 indicating metabolites from Pana extracts. Solid lines represent a series of molecular species differing by only two hydrogen atoms, and thus degrees of unsaturation. Dashed line represents metabolites with identical KMD, in this case differing in composition by C_2_H_4_ units.

Once these lists had been compiled after HMDB import, KMD were computed for each individual *m*/*z* value in each cultivar, then sorted according to their calculated KMD in Excel. This step, coupled with plotting KMD vs. nominal Kendrick mass for each cultivar, was essential for the annotation of several additional ionic formulas. The utility of Kendrick plots is highlighted by Figure 2, which is a Kendrick plot for the Pana leaf methanolic extract over the Kendrick nominal mass range of 550–750. Several features become apparent when displayed in this way that are valuable for annotation of complex mixtures. First, as shown by the solid lines, species which differ by two hydrogen atoms form diagonals with parallel slopes, enabling determination of many ionic formulas graphically when one member of the class is known. Second, horizontal lines represent chemical classes that differ only in the number of alkyl units. In metabolomics, this is often represented by ethylene (C_2_H_4_) differences common to fatty acids and acylglycerols; one example is shown in Figure 2 as a dashed line.

Let an example illustrate the utility of incorporating KMD analysis into the workflow. For Pana leaf extracts, there is a peak at *m*/*z* 333.20356 (observed in PI 567731 leaf extracts at *m*/*z* 333.20400). Based on entries in PubMed and considering only ionization by a proton, sodium ion or potassium ion, the peak at *m*/*z* 333.20356 could be C_16_H_24_N_6_O^+^, C_16_H_32_N_2_OS_2_^+^, or C_18_H_30_NaO_4_^+^; this last peak has a hit in the SoyCyc database of α,ω-9Z-octadecenedioc acid. KMD plots indicate help visualize peaks with two fewer hydrogens (C_18_H_28_NaO_4_^+^) and two more hydrogens (C_18_H_32_NaO_4_^+^). The peak corresponding to the formula C_18_H_28_O_4_ is not present in the SoyCyc database, indicating a new metabolite, while the peak corresponding to C_18_H_32_O_4_ is 9,10-12,13-diepoxyoctadecanoatic acid, which is present in SoyCyc. Likewise, after KMD, it is clear that peaks at *m*/*z* 333.18263 and 335.19826 are due to potassiated C_18_H_30_O_3_ and C_18_H_32_O_3_, respectively, from epoxy dienoic acids.

The net result after orthogonal KMD analysis yields assignment of more than 400 ionic formulas to the metabolites in the Pana leaf extracts (Supplementary Table S1), and more than 300 in the PI 567731 leaf extracts (Supplementary Table S2). Most of these formulas do not correlate with any compounds currently catalogued in SoyCyc. Thus, the combination of high-resolution MS and KMD analysis provides for identification of molecular formulas for unreported soybean metabolites. To date, application of KMD for the analysis of plant metabolites is sparse [37]; clearly, application of KMD after filtering *m*/*z* lists through databases considerably expands the total number of ionic formulas identified. Because many of these are identified for the first time, the results indicate that this process of filtering high mass accuracy spectra through databases for initial formula identification followed by KMD analysis generates an expanded inventory of leaf metabolites from different soybean cultivars.

### 3.4. Metabolic Pathways Analysis of Cultivars

Having matched molecular formulas to compounds in the SoyCyc and Human Metabolome Database, a list of the compounds which matched entries in SoyCyc was created for each cultivar. Each list (Pana, Supplementary Table S3; PI 567731, Supplementary Table S4) was an input which was then subjected to metabolic pathways analysis using the Pathway Covering tool as described in the Materials and Methods section.

Metabolic pathways analysis of the drought-susceptible Pana cultivar using a constant cost function and a minimal-cost set of metabolic pathways for *Glycine max* resulted in the calculated pathways indicated in Table 3. Eight distinct metabolic pathways were identified from the methanolic leaf extracts of the Pana cultivar: chlorophyll *a* degradation II, chlorophyll cycle, glycolipid desaturation, medicarpin conjugates interconversion, phytosterol biosynthesis, plant sterol biosynthesis II, superpathway of carotenoid biosynthesis, and violdelphin biosynthesis. Under the growing conditions used here, the leaves from the Pana cultivar were not under water-stress conditions; rather, they were under conventional irrigation conditions. Of particular interest, metabolites from the chlorophyll *a* degradation II and chlorophyll cycles were identified in a metabolomics study, of the Pana cultivar, although neither leaf age nor stress caused by drought treatment were found to vary in any statistically significant way for Pana cultivar leaf metabolites from these two pathways [14]. Nonetheless, the identification of additional pathways and compounds from them provide potential targets to examine the impact of drought on the Pana cultivar.

The metabolic pathways analysis for the drought-tolerant PI 567731 cultivar is shown in Table 4. Note that the pathways found in Pana are also identified in PI 567731 except for two: the medicaripin conjugates interconversion and violdelphin biosynthesis pathways. The chemical compounds from these common pathways are identical. Here, the growing conditions used do not induce water stress.

### 3.5. Implications on Previous Metabolomics Investigation of Drought Stress on Cultivars [14]

Our previous study found that levels of metabolites from the chlorophyll *a* degradation II cycle and the chlorophyll cycle detected in PI 567731 methanolic leaf extracts were quantitatively less impacted by drought-stress than the corresponding metabolites detected from the Pana methanolic leaf extracts [14]. This may be related the water preservation strategy employed by PI 567731, which exhibits the SW phenotype. Quantitative examination of compounds involved in the photosynthetic pathways are clearly warranted, as they provide potential targets for metabolomic profiling in different *Glycine max* cultivars; LC-MS should be employed to reduce matrix effects and control for different cationization agents.

Nonetheless, identification of several molecular formulas here emphasizes other metabolites in both the Pana and PI 567731 leaf extracts, which may reflect adaptation to drought beyond the chlorophyll pathways. Two of these metabolites are found in the glycolipid desaturation pathways noted in Table 3 and Table 4. Potassiated 1-18:3-2-18:3-monogalactosyldiacylglycerol was identified here, and is present in both cultivars as controls and after drought stress [14]. In drought-susceptible Pana, the relative level of this metabolite in young/old leaf extracts decreases from 0.820 to 0.380 after drought stress, while in PI 567731 this ratio increases from 0.455 to 1.537, indicating increased production of this metabolite upon drought stress. Potassiated 1-18:3-2-18:3-digalactosyldiacylglycerol is also identified here, and is present in both cultivars as controls but only in Pana after drought stress [14]. In drought-susceptible Pana, the relative level of this metabolite in young/old leaf extracts remains relatively constant, from 0.160 to 0.136 after drought stress. This metabolite is absent in PI 567731 after drought stress.

The sodiated bis(β-D-glucosyl) crocetin was identified and is present in both cultivars as controls and after drought stress [14]. In drought-susceptible Pana, relative levels of this metabolite in young/old leaf extracts increases from 0.599 to 0.879 after drought stress, while in the drought-tolerant PI 567731, these relative levels increase more significantly from 0.752 to 4.348. This metabolite is not involved in any of the pathways noted in Table 3 and Table 4. Also, the potassiated 3-β-D-galactosyl-sn-glycerol was identified; it is present in both cultivars as controls and after drought stress [14]. In drought-susceptible Pana, relative levels of this metabolite in young/old leaf extracts increases from 0.625 to 0.815 upon drought stress. In drought-tolerant PI567731, relative levels in young/old leaf extracts increases even more dramatically, from 0.632 to 4.373. While this metabolite is not involved in any of the pathways noted in Table 3 and Table 4, it is formed from the degradation of diacyl glycerols as noted above.

## 4. Conclusions

In this study, high-resolution mass spectrometry was used to identify hundreds of different molecular formulas as being present or absent from leaves of two soybean cultivars with different levels of tolerance to drought under well-watered conditions. An important component of this workflow involves KMD analysis. Upon KMD analysis of the data, coupled with graphical display of Kendrick mass plots, additional ionized molecular formulas were identified. One particular utility of the Kendrick mass plots was in identification of formulas differing in mass by only two hydrogen atoms, which are readily visualized; this indicates species which likely differ by one degree of unsaturation. Clearly, the use of KMD analysis to expand plant metabolite annotation is a methodology that should be more thoroughly considered. Finally, the *m*/*z* data, when filtered through SoyCyc, identified compounds that are key parts of metabolic pathways common to both the Pana and PI 567731 cultivars. These pathways include photosystem pathways involving chlorophyll, glycolipid desaturation, and biosynthetic pathways to generate sterols, phytosterols, and carotenoids. Pathway analysis provides additional viable targets for future study of impacts of abiotic stressors and drought tolerance to soybean plant metabolite expression. Several metabolites including chlorophylls, pheophytins, mono- and diacylglycerols, cycloeucalenone, squalene, and plastoquinones are interesting molecular targets, and identified pathways include those for anabolism and catabolism of chlorophyll, glycolipid desaturation, and biosynthesis of phytosterols, plant sterols, and carotenoids. When applied to previous results [14], metabolites involved in glycolipid desaturation show alterations due to drought stress, making them particularly attractive molecular targets for future studies. Such insights may generate focus upon the molecular mechanisms by which certain cultivars adapt to stressors in comparison to other cultivars, and ultimately the utility for metabolomic analysis of plant stress to guide phenotypic expression of plant traits for adaptation.

## Figures and Tables

**Table 1:** Measured *m*/*z* for ionic forms of metabolites uniquely detected in Pana leaf methanolic extracts.

Measured *m*/*z*	Ion Formula	Mass Error (ppm)	Possible Identification
309.20358	C_16_H_30_NaO_4_	−0.16	Hexdecanedioic acid
325.17755	C_19_H_26_NaO_3_	0.14	Carlactone
351.17563	C_18_H_32_KO_2_S	0.49	Carboxylic acid class
353.22983	C_18_H_34_NaO_5_	−0.04	Stearic acid isomers
359.01734	C_15_H_12_KO_8_	−2.59	Carboxylic acid class
365.06327	C_15_H_18_KO_8_	−0.15	Carboxylic acid class
367.10843	C_10_H_23_O_14_	0.54	Carboxylic acid class
395.17342	C_21_H_28_KN_2_O_3_	0.68	Galatopinitol
435.25066	C_23_H_40_KO_5_	−0.17	5-isomers
471.25074	C_26_H_40_KO_5_	0.02	Glucoside class
497.18664	C_20_H_33_O_14_	−0.32	3 isomers
585.37007	C_40_H_50_NaO_2_	−0.40	15-cis-phytoene
609.27067	C_29_H_46_NaO_10_S	0.46	3 isomers
647.46492	C_46_H_66_NaO_2_	−0.40	Epoxypheophorbide *a*
649.18953	C_29_H_38_KO_14_	0.33	Glucoside class
675.49608	C_51_H_96_KO_6_	0.05	2 isomers
741.57946	C_17_H_25_NaNO_6_	0.10	Menaquinol-8
771.60506	C_56_H_96_KO_3_	0.01	34:5-monoglactosyldiacylglycerol
893.55467	C_55_H_74_NaN_4_O_5_	−0.53	Pheophytin *a*
907.52140	C_55_H_71_MgN_4_O_6_	−0.50	Chlorophyll *b*
923.50859	C_55_H_72_KN_4_O_6_	0.27	Pheophytin *b*
945.47643	C_55_H_70_KMgN_4_O_6_	−1.38	Chlorophyll *b*

**Table 2:** Measured *m*/*z* for ionic forms of metabolites uniquely detected in PI 567731 leaf methanolic extracts.

Measured *m*/*z*	Ion Formula	Mass Error (ppm)	Possible Identification
277.08988	C_9_H_18_NaO_8_	1.77	3-β-D-galactosyl-sn-glycerol
481.36516	C_30_H_50_NaO_3_	−0.12	Soyasapogenol B
527.15854	C_18_H_92_NaO_16_	0.54	Trisaccharide class
543.13251	C_18_H_92_KO_16_	0.74	Trisaccharide class
771.60547	C_50_H_84_KO_3_	0.34	Plastoquinone

**Table 3:** Metabolic pathways analysis for metabolites detected in Pana cultivar methanolic leaf extracts.

Pathway	Covered Compounds
Chlorophyll *a* degradation II	Pheophytin *a* Chlorophyll *a*
Chlorophyll cycle	Chlorophyll *a* Chlorophyll *b*
Glycolipid desaturation	1-18:2-2-18:2-monogalactosyldiacylglycerol 1-18:3-2-18:3-monogalactosyldiacylglycerol 1-18:3-2-18:3-digalactosyldiacylglycerol
Medicarpin conjugates interconversion	Medicarpin-3-*O*-glucoside
Phytosterol biosynthesis	Cycloeucalenone
Plant sterol biosynthesis II	Squalene
Superpathway of carotenoid biosynthesis	Antheraxanthin Plastoquinone 15-*cis*-phytoene
Violdelphin biosynthesis	Delphinidin-3-*O*-rutioside-7-*O*-glucoside

**Table 4:** Metabolic pathways analysis for metabolites detected in PI 567731 cultivar methanolic leaf extracts.

Pathway	Covered Compounds
Chlorophyll *a* degradation II	Pheophytin *a* Chlorophyll *a*
Chlorophyll cycle	Chlorophyll *a* Chlorophyll *b*
Glycolipid desaturation	1-18:2-2-18:2-monogalactosyldiacylglycerol 1-18:3-2-18:3-monogalactosyldiacylglycerol 1-18:3-2-18:3-digalactosyldiacylglycerol
Phytosterol biosynthesis	Cycloeucalenone
Plant sterol biosynthesis II	Squalene
Superpathway of carotenoid biosynthesis	Antheraxanthin Plastoquinone 15-*cis*-phytoene

## Data Availability

Processed data files are provided as Supplementary Tables S1 and S2. The original data presented in this study are openly available in Dryad with a unique digital object identifier (DOI): https://doi.org/10.5061/dryad.np5hqc046 accessed on 18 January 2025.

## Supplementary Materials

The following supporting information can be downloaded at: https://www.mdpi.com/article/10.3390/metabo15020073/s1, Table S1: Ionic formulas and KMD analysis for species in Pana leaf extracts; Table S2: Ionic formulas and KMD analysis for species in PI 567731 leaf extracts; Table S3: List of compounds from Pana leaf extracts for pathways analysis; Table S4: List of compounds from PI 567731 leaf extracts for pathways analysis.

## References

[1] Lesk C., Rowhani P., Ramankutty N. (2016). Influence of extreme weather disasters on global crop production. Nature.

[2] Frederick J.R., Camp C.R., Bauer P.J. (2001). Drought-Stress Effects on Branch and Mainstem Seed Yield and Yield Components of Determinate Soybean. Crop Sci..

[3] Das A., Rushton P.J., Rohila J.S. (2017). Metabolomic Profiling of Soybeans (*Glycine max* L.) Reveals the Importance of Sugar and Nitrogen Metabolism under Drought and Heat Stress. Plants.

[4] Ye H., Song L., Schapaugh W.T., Ali L., Sinclair T.R., Riar M.K., Raymond R.N., Li Y., Vuong T., Valliyodan B. (2020). The importance of slow canopy wilting in drought tolerance in soybean. J. Exp. Bot..

[5] Pathan S.M., Lee J.-D., Sleper D.A., Fritschi F.B., Sharp R.E., Carter T.E., Nelson R.L., King C.A., Schapaugh W.T., Ellersieck M.R. (2014). Two Soybean Plant Introductions Display Slow Leaf Wilting and Reduced Yield Loss under Drought. J. Agron. Crop Sci..

[6] Du Y.L., Zhao Q., Chen L.R., Yao X.D., Zhang H.J., Wu J.J., Xie F.T. (2020). Effect of Drought Stress during Soybean R2–R6 Growth Stages on Sucrose Metabolism in Leaf and Seed. Int. J. Mol. Sci..

[7] Ncube E., Mohale K., Nogemane N. (2022). Metabolomics as a Prospective Tool for Soybean (*Glycine max*) Crop Improvement. Curr. Issues Mol. Biol..

[8] Silva E., Belinato J.R., Porto C., Nunes E., Guimaraes F., Meyer M.C., Pilau E.J. (2021). Soybean Metabolomics Based in Mass Spectrometry: Decoding the Plant’s Signaling and Defense Responses under Biotic Stress. J. Agric. Food Chem..

[9] Jervis J., Kastl C., Hildreth S.B., Biyashev R., Grabau E.A., Saghai-Maroof M.A., Helm R.F. (2015). Metabolite Profiling of Soybean Seed Extracts from Near-Isogenic Low and Normal Phytate Lines Using Orthogonal Separation Strategies. J. Agric. Food Chem..

[10] Komatsu S., Nakamura T., Sugimoto Y., Sakamoto K. (2014). Proteomic and Metabolomic Analyses of Soybean Root Tips Under Flooding Stress. Protein Pept. Lett..

[11] Wang X.Y., Li Y.P., Wang X.J., Li X.M., Dong S.K. (2022). Physiology and metabonomics reveal differences in drought resistance among soybean varieties. Bot. Stud..

[12] Velickovic D., Agtuca B.J., A Stopka S., Vertes A., Koppenaal D.W., Pasa-Tolic L., Stacey G., Anderton C.R. (2018). Observed metabolic asymmetry within soybean root nodules reflects unexpected complexity in rhizobacteria-legume metabolite exchange. ISME J..

[13] Yilmaz A., Rudolph H.L., Hurst J.J., Wood T.D. (2016). High-Throughput Metabolic Profiling of Soybean Leaves by Fourier Transform Ion Cyclotron Resonance Mass Spectrometry. Anal. Chem..

[14] Zemaitis K.J., Ye H., Nguyen H.T., Wood T.D. (2021). Direct Infusion Metabolomics of the Photosystem and Chlorophyll Related Metabolites within a Drought Tolerant Plant Introduction of *Glycine max*. Metabolites.

[15] Bragagnolo F.S., Funari C.S., Ibanez E., Cifuentes A. (2021). Metabolomics as a Tool to Study Underused Soy Parts: In Search of Bioactive Compounds. Foods.

[16] Kendrick E. (1963). A Mass Scale Based on CH_2_ = 14.0000 for High Resolution Mass Spectrometry of Organic Compounds. Anal. Chem..

[17] Hughey C.A., Hendrickson C.L., Rodgers R.P., Marshall A.G. (2001). Elemental Composition Analysis of Processed and Unprocessed Diesel Fuel by Electrospray Ionization Fourier Transform Ion Cyclotron Resonance Mass Spectrometry. Energy Fuels.

[18] Hughey C.A., Hendrickson C.L., Rodgers R.P., Marshall A.G., Qian K. (2001). Kendrick Mass Defect Spectrum: A Compact Visual Analysis for Ultrahigh-Resolution Broadband Mass Spectra. Anal. Chem..

[19] Chainet F., Ponthus J., Lienemann C.-P., Courtiade M., Donard O.F.X. (2012). Combining Fourier Transform-Ion Cyclotron Resonance/Mass Spectrometry Analysis and Kendrick Plots for Silicon Speciation and Molecular Characterization in Petroleum Products at Trace Levels. Anal. Chem..

[20] Cody R.B., Fouquet T. (2018). Resolution-Enhanced Kendrick Mass Defect Analysis of Polycyclic Aromatic Hydrocarbons and Fullerenes in the Diffusion Flame from a Butane Torch. J. Am. Soc. Mass Spectrom..

[21] Spiegel M.T., Anthony I.G.M., Brantley M.R., Hassell A., Farmer P.J., Solouki T. (2018). Reactivities of Aromatic Protons in Crude Oil Fractions toward Br_2_ Tagging for Structural Characterization by Nuclear Magnetic Resonance and Electron Paramagnetic Resonance Spectroscopy and Mass Spectrometry. Energy Fuels.

[22] Sato H., Nakamura S., Teramoto K., Sato T. (2014). Structural Characterization of Polymers by MALDI Spiral-TOF Mass Spectrometry Combined with Kendrick Mass Defect Analysis. J. Am. Soc. Mass Spectrom..

[23] Fouquet T., Sato H. (2016). Convenient visualization of high-resolution tandem mass spectra of synthetic polymer ions using Kendrick mass defect analysis—The case of polysiloxanes. Rapid Commun. Mass Spectrom..

[24] Cody R.B., Fouquet T. (2017). Paper spray and Kendrick mass defect analysis of block and random ethylene oxide/propylene oxide copolymers. Anal. Chim. Acta.

[25] Fouquet T., Sato H. (2017). Improving the Resolution of Kendrick Mass Defect Analysis for Polymer Ions with Fractional Base Units. Mass Spectrom..

[26] Fouquet T., Sato H. (2017). Extension of the Kendrick Mass Defect Analysis of Homopolymers to Low Resolution and High Mass Range Mass Spectra Using Fractional Base Units. Anal. Chem..

[27] Fouquet T., Shimada H., Maeno K., Ito K., Ozeki Y., Kitagawa S., Ohtani H., Sato H. (2017). High-resolution Kendrick Mass Defect Analysis of Poly(ethylene oxide)-based Non-ionic Surfactants and Their Degradation Products. J. Oleo Sci..

[28] Cody R.B., Fouquet T. (2018). “Reverse Kendrick Mass Defect Analysis”: Rotating Mass Defect Graphs to Determine Oligomer Compositions for Homopolymers. Anal. Chem..

[29] Fouquet T.N.J., Cody R.B., Ozeki Y., Kitagawa S., Ohtani H., Sato H. (2018). On the Kendrick Mass Defect Plots of Multiply Charged Polymer Ions: Splits, Misalignments, and How to Correct Them. J. Am. Soc. Mass Spectrom..

[30] Korf A., Fouquet T., Schmid R., Hayen H., Hagenhoff S. (2020). Expanding the Kendrick Mass Plot Toolbox in MZmine 2 to Enable Rapid Polymer Characterization in Liquid Chromatography-Mass Spectrometry Data Sets. Anal. Chem..

[31] He H., Conrad C.A., Nilsson C.L., Ji Y.J., Schaub T.M., Marshall A.G., Emmett M.R. (2007). Method for Lipidomic Analysis: p53 Expression Modulation of Sulfatide, Ganglioside, and Phospholipid Composition of U87 MG Glioblastoma Cells. Anal. Chem..

[32] Lerno L.A., German J.B., Lebrilla C.B. (2010). Method for the Identification of Lipid Classes Based on Referenced Kendrick Mass Analysis. Anal. Chem..

[33] Korf A., Vosse C., Schmid R., Helmer P.O., Jeck V., Hayen H. (2018). Three-dimensional Kendrick mass plots as a tool for graphical lipid identification. Rapid Commun. Mass Spectrom..

[34] Chevalier M., Ricart E., Hanozin E., Pupin M., Jacques P., Smargiasso N., De Pauw E., Lisacek F., Leclere V., Flahaut C. (2019). Kendrick Mass Defect Approach Combined to NORINE Database for Molecular Formula Assignment of Nonribosomal Peptides. J. Am. Soc. Mass Spectrom..

[35] Blanc L., Ferraro G.B., Tuck M., Prideaux B., Dartois V., Jain R.K., Desbenoit N. (2021). Kendrick Mass Defect Variation to Decipher Isotopic Labeling in Brain Metastases Studied by Mass Spectrometry Imaging. Anal. Chem..

[36] Muller W.H., Verdin A., Kune C., Far J., De Pauw E., Malherbe C., Eppe G. (2021). Dual-polarity SALDI FT-ICR MS imaging and Kendrick mass defect data filtering for lipid analysis. Anal. Bioanal. Chem..

[37] Zarev Y., Popova P., Foubert K., Ionkova I., Pieters L. (2021). Comparative LC-MS analysis of tropolone alkaloids from in vitro cultures and native sources of *Gloriosa superba* by Kendrick mass defect plots. Phytochem. Anal..

[38] Richardson L.T., Neumann E.K., Caprioli R.M., Spraggins J.M., Solouki T. (2022). Referenced Kendrick Mass Defect Annotation and Class-Based Filtering of Imaging MS Lipidomics Experiments. Anal. Chem..

[39] Rivas-Ubach A., Liu Y., Bianchi T.S., Tolic N., Jansson C., Pasa-Tolic L. (2018). Moving beyond the van Krevelen Diagram: A New Stoichiometric Approach for Compound Classification in Organisms. Anal. Chem..

[40] Brown A.V., I Conners S., Huang W., Wilkey A.P., Grant D., Weeks N.T., Cannon S.B., A Graham M., Nelson R.T. (2021). A new decade and new data at SoyBase, the USDA-ARS soybean genetics and genomics database. Nucleic Acids Res..

[41] Wishart D.S., Tzur D., Knox C., Eisner R., Guo A.C., Young N., Cheng D., Jewell K., Arndt D., Sawhney S. (2007). HMDB: The Human Metabolome Database. Nucleic Acids Res..

[42] Karp P.D., E Midford P., Billington R., Kothari A., Krummenacker M., Latendresse M., Ong W.K., Subhraveti P., Caspi R., Fulcher C. (2021). Pathway Tools version 23.0 update: Software for pathway/genome informatics and systems biology. Briefings Bioinform..

[43] Fang C., Fernie A.R., Luo J. (2019). Exploring the Diversity of Plant Metabolism. Trends Plant Sci..

