# Supplementary material for: Chemical Informatics Combined with Kendrick Mass Analysis to Enhance Annotation and Identify Pathways in Soybean Metabolomics
Source: Metabolites. 2025 Jan 24;15(2):73. doi: 10.3390/metabo15020073 (PMC11857611; doi:10.3390/metabo15020073)
Supplement: Supplementary file 1 [file metabolites-15-00073-s001.zip › Table S4.pdf]

Table S4: List of Compounds from PI 567731 Leaf Extracts for Pathways Analysis

1-[18-hydroxyoeoyl]-2-[18-hydroxy-linoleoyl]-sn-glycerol

1-18:2-2-18:2-monogalactosyldiacylglycerol

1-18:3-2-18:3-digalactosyldiacylglycerol

1-18:3-2-18:3-monogalactosyldiacylglycerol

15-cis-phytoene

3-beta-D-galactosyl-sn-glycerol

plastoquinone

antheraxanthin

bis(beta-D-glucosyl) crocetin

chlorophyll a

chlorophyll b

cyanidin-3-O-beta-D-galactoside

cycloeucalenone

demethylmenaquinol-8

echinenone

pelargonidin-3-O-rutinoside-5-O-beta-D-glucoside

pheophytin a

soyasoponin B

squalene

ubiquinol-9
